# Pan-Spinal Epidural Abcess: Multidisciplinary Management of a Rare and Life-Threatening Condition

**DOI:** 10.7759/cureus.101533

**Published:** 2026-01-14

**Authors:** Pedro Oliveira, Maria E Batista, Miguel Barbosa, Joana Vaz, André Borges

**Affiliations:** 1 Department of Critical Care Medicine, Hospital Curry Cabral, Unidade Local de Saúde de São José, Lisbon, PRT; 2 Department of Critical Care Medicine, Unidade Local de Saúde do Médio Tejo, Abrantes, PRT

**Keywords:** case report, hyperbaric oxygen therapy, laminectomy, pan-spinal abscess, spinal epidural abscess

## Abstract

We report the case of a 28-year-old male with a history of intravenous drug use who presented with low back pain, progressive paraparesis, fever, and respiratory distress. MRI revealed an extensive epidural abscess from C1 to the sacrum with associated paraspinal muscle collections. Within 24 hours, he underwent staged cervicothoracic and thoracolumbar laminectomies with drainage of purulent material, which cultured methicillin-resistant *Staphylococcus aureus*. The patient received targeted intravenous vancomycin, completed 10 sessions of hyperbaric oxygen therapy, and required a second surgical drainage for residual abscess. Progressive neurological recovery followed, and he was successfully extubated after eight days. Spinal epidural abscess is an infrequent but potentially life-threatening infection. Pan-spinal or holo-spinal involvement, in which the abscess extends throughout the entire neuraxis, is exceedingly rare and carries high morbidity and mortality. This case highlights the critical importance of maintaining a high index of suspicion, initiating prompt empirical antibiotic therapy, and performing timely multidisciplinary surgical intervention.

## Introduction

Spinal epidural abscess (SEA) is a suppurative infection characterized by the accumulation of pus in the epidural space [1,2]. The incidence has doubled over the past decade, with recent meta-analyses reporting prevalence rates ranging from 0.18 to 1.96 per 10,000 hospital admissions. The prevalence is higher in males (2:1 male-to-female ratio) and is most common between the sixth and seventh decades of life [1]. Risk factors are categorized as either local or systemic [1]. Systemic factors include diabetes mellitus, class III obesity, chronic steroid use, intravenous drug use, alcoholism, chronic renal disease, malignancy, and HIV infection. Local predisposing factors encompass recent spinal trauma, minimally invasive surgical procedures (e.g., intrathecal drug administration, catheter placement), or other surgical interventions [1,2].

SEA can occur at any spinal level. The thoracic and lumbar regions are the most frequently affected; however, cervical epidural abscesses are less common, accounting for 18-36% of cases [2]. These abscesses are often associated with high morbidity and mortality, particularly when neurological impairment is present [1]. Even rarer are pan-spinal or holo-spinal infections, documented only in individual case reports; these infections can spread across multiple segments between the cervical and lumbosacral regions. Such cases typically present with rapid neurological deterioration across several levels, diffuse deficits, delayed diagnosis, and challenging source control, ultimately leading to poorer clinical outcomes [2].

The therapeutic gold standard is early diagnosis and surgical control of the source, followed by intravenous antibiotics. The clinical presentation varies with location and extent and may include fever, localized pain, neurologic deficits (e.g., sphincter dysfunction, paraplegia), or meningeal irritation (neck stiffness, Lhermitte’s sign) [1-3]. Although early changes are seen on CT, MRI is the diagnostic gold standard due to its high sensitivity and specificity. The classic findings are a mass-effect lesion that is hypointense on T1 and heterogeneously hyperintense on T2, with contrast enhancement. Etiologic recognition nearly invariably requires blood cultures and culture of material drained [1,2,4].

Treatment remains controversial; however, emergency surgical drainage is strongly recommended, particularly in cases of unsuccessful conservative management, deteriorating neurological deficits, spinal instability, abscesses exceeding 2.5 cm in size, ischemia, compression, or sepsis [1]. Intravenous broad-spectrum antibiotics should be initiated and subsequently adjusted based on culture and sensitivity results, typically for four to six weeks. Multilevel laminectomy (extensive decompression) may be necessary for pan-spinal disease [1,2,4]. Schulz et al. observed a favorable neurological outcome after applying skip laminectomies with catheter-based epidural irrigation [5]. Hyperbaric oxygen therapy has been reported in some cases as an adjuvant to surgical source control during antibiotic therapy [6].

We present a case of a patient with a pan-SEA who was diagnosed and managed promptly through swift, coordinated efforts among the departments of intensive care, neuroradiology, and neurosurgery.

## Case presentation

In June 2025, a 28-year-old male who had been residing in Portugal for the past four years, with current drug use (cannabis, ecstasy, 3-MMC, and intravenous methamphetamine) and no other health issues or regular medications, presented to the emergency department with a one-week history of low back pain and ascending weakness in the lower limbs. The condition had worsened over the past 48 hours and is associated with fever, neck stiffness, and shortness of breath.

Upon admission, the patient presented with confusion (Glasgow Coma Scale score of 14), bilateral lower-limb hypoesthesia without a clearly defined sensory level, severe weakness with absence of antigravity strength, and flaccid tone with no resistance to passive movement at the lower-limb joints. Plantar responses were flexor bilaterally. Neck stiffness was present. The patient was febrile at 38.6°C, experiencing polypnea with the use of accessory muscles, and had an SpO₂ of 95% while receiving a non-rebreather mask at 15 L/min. Lung auscultation revealed normal sounds. Arterial blood gas analysis indicated partial respiratory failure (pH 7.365, pCO₂ 42.2 mmHg, pO₂ 68.2 mmHg, HCO₃⁻ 24.1 mmol/L). Symptoms developed following attendance at a slamming party associated with prolonged intravenous drug use and unprotected sexual activity. Laboratory investigations demonstrated significant inflammation, with a white blood cell count of 30.98 × 10⁹/L (89.1% neutrophils), CRP at 436 mg/L, and procalcitonin at 15 ng/mL. Renal impairment was evidenced by urea levels of 70 mg/dL and creatinine of 1.77 mg/dL. Indicators of myositis included a creatine kinase level of 1464 U/L and myoglobin of 5592 ng/mL. Toxicology screening and viral serology, including HIV, yielded negative results. Chest radiography was within normal limits. CT scans of the chest, abdomen, and pelvis revealed a consolidative lesion in the anterior segment of the right upper lobe, severe bladder distension causing bilateral upper urinary tract ectasia, and generalized colonic dilation. A normal brain CT scan was noted, while cervical spine imaging indicated posterior epidural space widening of uncertain significance.

**Figure 1:**
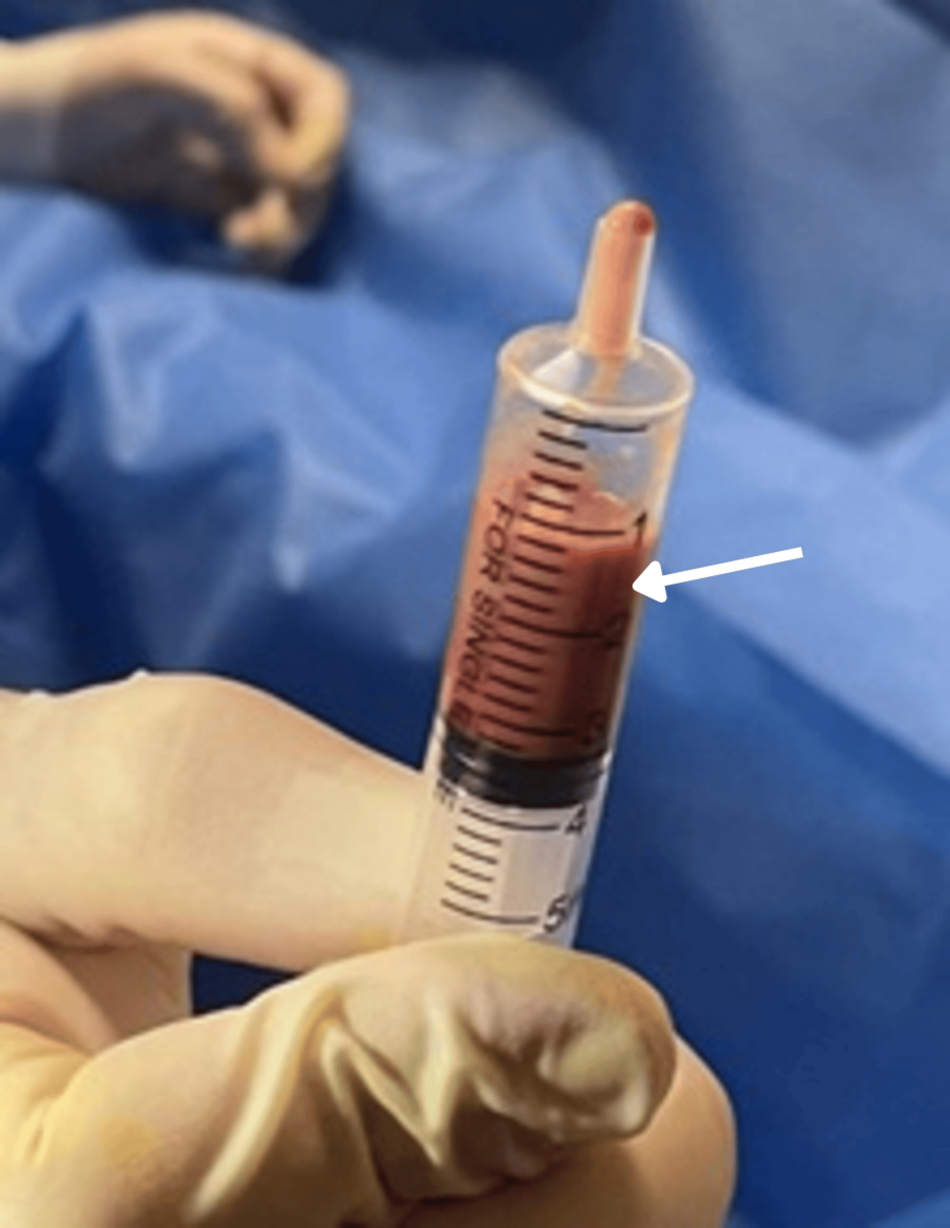
CSF sample Macroscopic appearance of CSF obtained via lumbar puncture, demonstrating a turbid, purulent quality (white arrow). CSF: cerebrospinal fluid

The patient presented with severe partial respiratory failure and was admitted to the intensive care unit (ICU). During the initial six hours, the patient experienced rapid deterioration characterized by deficits including ascending areflexic and hypotonic paraplegia, upper limb paraparesis graded as III in strength, bilateral symmetric lower limb hypesthesia without a distinct sensory level, areflexia, cervicalgia, neck stiffness, and a positive test for Lhermitte’s phenomenon. The clinical course progressed to respiratory fatigue, necessitating intubation and the initiation of mechanical ventilation. Given the presence of cervical rigidity and a probable cervical spine condition, a difficult airway protocol was implemented to minimize cervical spine movement, use video laryngoscopy, and perform a local rapid sequence induction. Empiric therapy comprising ceftriaxone, vancomycin, acyclovir, and dexamethasone was commenced to address suspected meningoencephalitis. Lumbar puncture yielded 3 mL of turbid, purulent cerebrospinal fluid (CSF) for culture (Figure 1).

**Figure 2:**
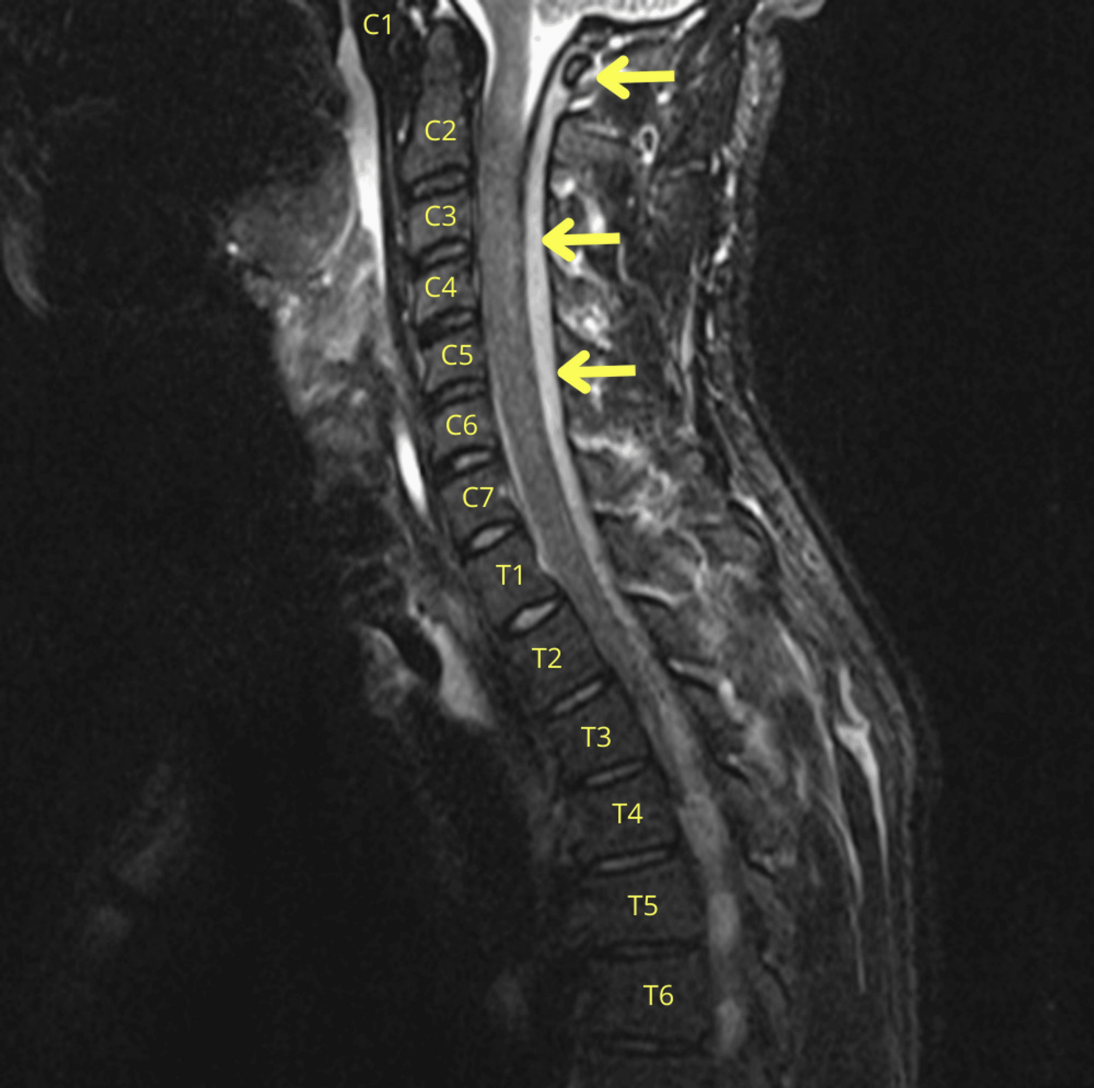
Sagittal cervical MRI scan T2-weighted images revealing extensive posterior-lateral epidural collection extending continuously from C1 to the sacrum (up to S2). Maximal thickness is ~6 mm at the cervical level (yellow arrows). MRI: magnetic resonance imaging

**Figure 3:**
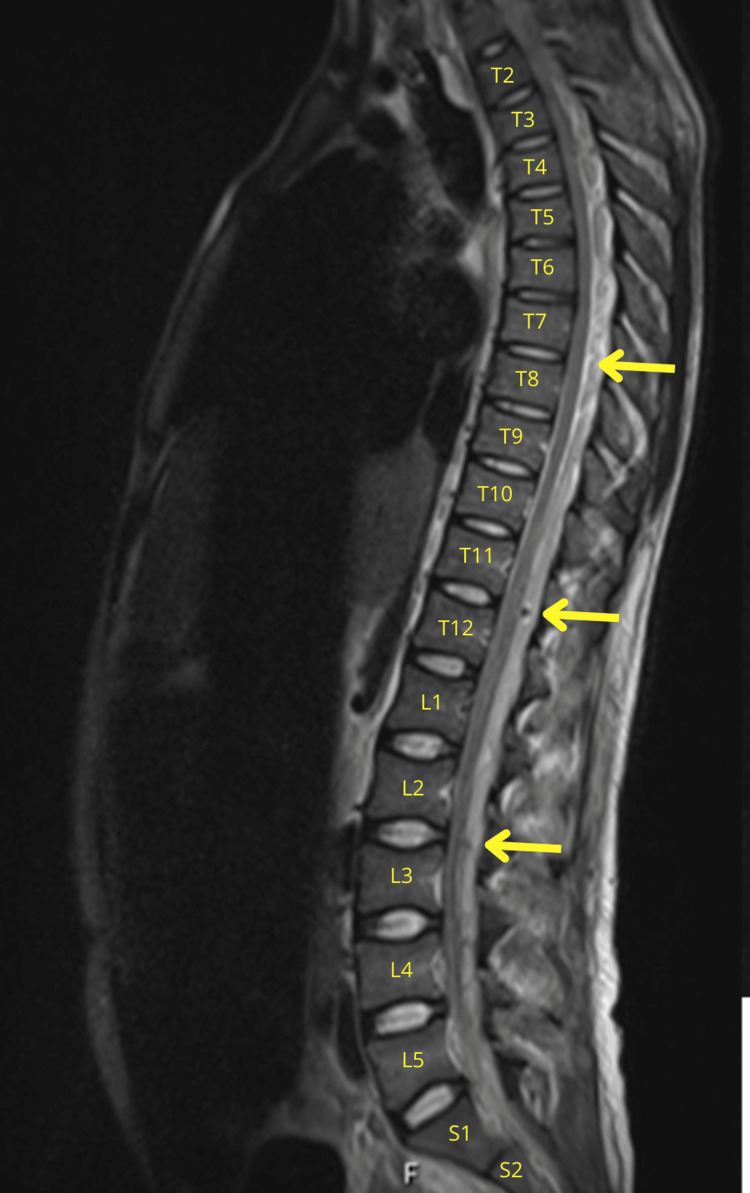
Sagittal thoraco-lumbar MRI scan T2-weighted images reveal an extensive posterior-lateral epidural collection extending continuously up to S2, with maximal thickness ~10 mm at thoracic and lumbar levels (yellow arrows). MRI: magnetic resonance imaging

The neurological findings, combined with the macroscopic appearance of the CSF, raised concerns of spinal cord compression. MRI of the entire spine demonstrated a continuous posterior and posterolateral epidural abscess extending from C1 to S2, isointense on T1-weighted images and heterogeneously hyperintense on T2-weighted images, with peripheral contrast enhancement. Maximal thickness was approximately 6 mm at the cervical level and up to 10 mm at the thoracic and lumbar levels, causing significant spinal canal compromise (Figures 2-3). Associated rim-enhancing paraspinal muscle collections and inflammatory changes from T10-T11 to S2 were also present, without evidence of vertebral osteomyelitis.

**Figure 4:**
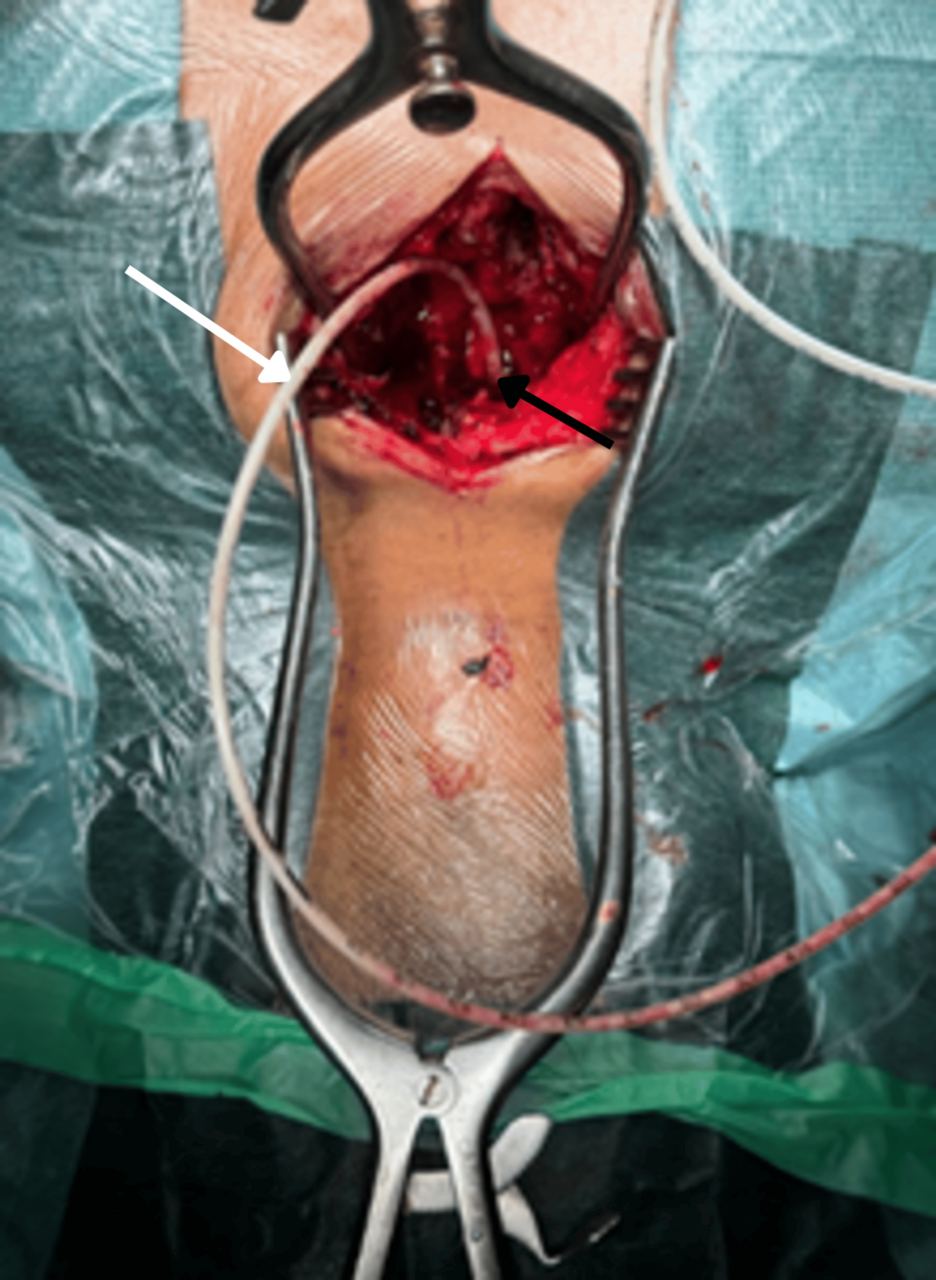
Dorsolombar laminectomy Intraoperative view during thoracolumbar laminectomy (black arrow). An intraepidural cannula (white arrow) is used to aspirate purulent material from the epidural space. This minimally invasive technique facilitated drainage across multiple levels.

**Figure 5:**
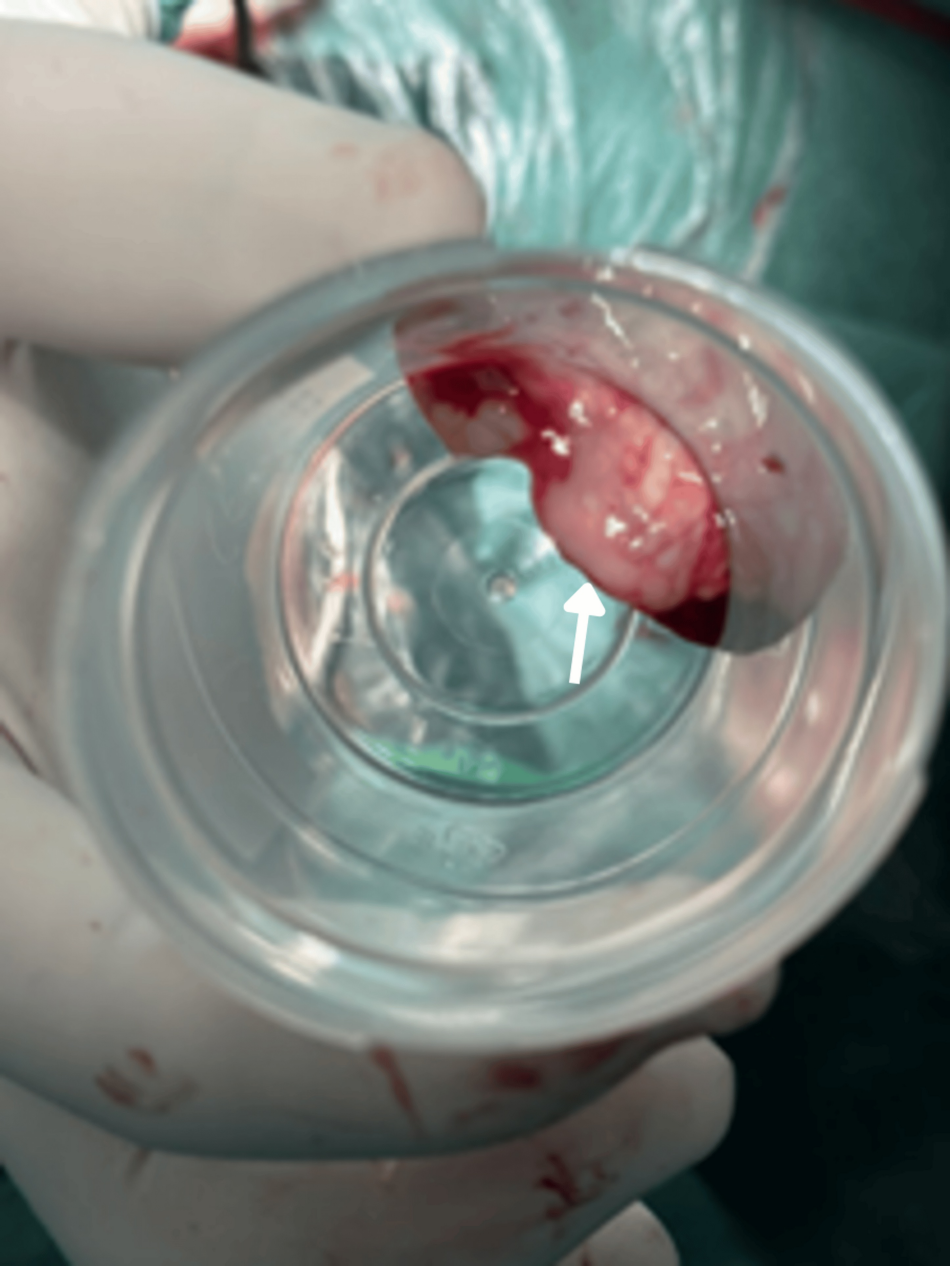
Pus collected during laminectomy Pus collected during thoracolumbar laminectomy (white arrow). Culture plate demonstrating growth of MRSA from the drained epidural pus. This confirmed the pathogen and guided targeted antibiotic therapy with vancomycin. MRSA: methicillin-resistant *Staphylococcus aureus*

The patient was managed within 24 hours of admission with laminectomies at the cervicothoracic and thoracolumbar junctions and evacuation of purulent material via an intracanal cannula, with samples sent for microbiology (Figures 4-5). All cultures on the specimen were methicillin-resistant *Staphylococcus aureus* (MRSA). Ceftriaxone and acyclovir were discontinued, and specific therapy with vancomycin was continued. The transesophageal echocardiogram was devoid of valvular or chamber abnormality.

During the ICU stay, the patient underwent 10 sessions of hyperbaric oxygen therapy performed at an external military institution. Detailed information on the hyperbaric protocol was unavailable because the treatment was administered outside our center. Despite the controversial role of adjunctive hyperbaric oxygen therapy in SEA and the logistical challenges associated with transporting a mechanically ventilated patient with spinal cord compression, the multidisciplinary team decided to proceed with this therapy. This decision was based on the severity of the disease, characterized by extensive abscess formation along the entire spinal axis, and the intraoperative inability to completely remove all infected material.

One week later, a repeat MRI demonstrated some evacuation of the abscess; however, an ongoing collection remained at the lower thoracic level, along with cord lesions bilaterally at C2-C3 and on the left at T2-T3. The patient subsequently underwent reintervention, during which additional drainage was performed through the existing dorsolumbar laminectomy incision and a lower lumbar laminectomy.

Following successful source control and targeted antibiotic therapy, the patient was afebrile at 48 hours, with declining inflammatory markers, and was extubated on day 8 (Table 1). Neurologically, there was notable improvement, with partial recovery of lower-limb strength (grade II on the right, grade I on the left), preserved light touch and thermal-pain sensation up to T8, and intact upper-limb function, though sphincter incontinence persisted. On the 13th day, the patient was transferred to the Infectious Diseases ward for continued antibiotic therapy and intensive rehabilitation.

**Table 1:** Laboratory values at admission and during hospitalization At admission, the patient had elevated inflammatory parameters, with CRP 436.5 mg/dL and procalcitonin 15.40 ng/mL, rhabdomyolysis with myoglobin of 445 ng/mL, and an acute renal lesion with low urine output. During the 7 days of hospitalization, we observed a reduction in inflammatory parameters, a normal myoglobin level on day 7, and resolution of the acute renal lesion. CRP: C-reactive protein

Parameter (unit)	Reference range	Admission	Day 2	Day 3	Day 7
WBC (x10⁹/L)	4.5-11.0	30.98	28.80	25.13	15.67
Neutrophils (x10⁹/L)	2.0-8.5	27.61	25.95	23.35	14.13
CRP (mg/dL)	<5.0	436.5	367.8	173.6	140.6
Procalcitonin (ng/mL)	<0.05	15.40	11.80	9.20	1.69
Mioglobina (ng/mL)	28.0-72	445	1035	220	150
Creatinine (mg/dL)	0.67-1.17	1.77	1.24	1.09	0.79
Urea (mg/dL)	16.6-48.5	70	65	75	52

## Discussion

SEA is an uncommon condition, more frequently observed in older males. The primary risk factors include acquired immunosuppression, intravenous drug use, alcoholism, and traumatic or iatrogenic breaches of the epidural space [1]. Our patient, despite being young, reported a history of repeated intravenous drug use, one of the most significant risk factors associated with SEA. Although co-infection with HIV or HCV is typical in this demographic, serologies were negative in this case. The most probable origin was local inoculation at injection sites, subsequently leading to hematogenous dissemination to the neuraxial spine and paraspinal muscles, as confirmed by MRI.

Although gram-negative bacilli are more frequently isolated among intravenous drug users, the most common pathogen remains *Staphylococcus aureus* [1-4], with MRSA identified in this patient. No evidence of vertebral osteomyelitis or other adjacent foci was observed. SEA can occur at any level within the spine; however, cervical localization is the rarest and is associated with the poorest functional and vital prognosis [1,2,4]. This case is notable for both the severity, characterized by holospinal spread, and its rapid progression and multilevel spinal neurological deficits.

Within a one-week period, the patient presented with ascending areflexic paraplegia, upper limb paresis, and symmetric sensory impairment lacking a definitive sensory level, accompanied by meningeal signs characteristic of cervical involvement. These clinical features, along with persistent fever and elevated inflammatory markers, strongly indicate an infectious etiology. The patchy distribution of neurological deficits across various dermatomes and myotomes, as well as sphincter dysfunction, suggested spinal cord compression, which was subsequently confirmed via MRI.

Early diagnosis was achieved in this patient within 24 hours of hospitalization. However, diagnosis is often delayed because neurological impairments may mimic cerebral or peripheral nervous system disorders [1,4]. Additionally, progression to septic shock and multi-organ failure may complicate the recognition process. The observation of Lhermitte’s sign served as an important indicator supporting the diagnosis of spinal cord compression.

MRI is the definitive diagnostic tool for identifying and planning the surgical approach, demonstrating characteristic contrast-enhancing epidural collections [1,4]. CT is occasionally employed as an adjunct when MRI is unavailable and provides less definitive information [4]. Empiric broad-spectrum antibiotics targeting common central nervous system pathogens should be administered promptly, ideally after obtaining cultures [1-4]. The administration of focused intravenous vancomycin resulted in clinical, laboratory, and radiographic resolution upon MRSA diagnosis.

Surgical intervention for source control is generally recommended in cases of neurological deterioration, abscesses exceeding 2.5 cm, spinal cord compression, or sepsis [1]. Our patient met all the aforementioned criteria and therefore underwent staged cervicothoracic and thoracolumbar laminectomies with drainage. The use of an intraepidural cannula to facilitate maximal evacuation of pus was innovative and minimally documented in the treatment of pan-spinal SEA [5]. The need for subsequent surgical procedures to address residual collections underscores the technical challenges of achieving complete evacuation in extensive cases.

Adjunctive hyperbaric oxygen therapy, an off-label treatment, was administered in this case, supported by prior reports suggesting potential benefits in reducing infectious burdens and neurologic deficits [7]. Potential mechanisms, such as enhanced leukocyte function, direct bactericidal effects on anaerobes, and improved tissue oxygenation, are thought to play a role in local infection control, angiogenesis stimulation, and tissue repair [7].

Patients with acute spinal cord injury, especially at cervical or high thoracic levels, are at high risk of respiratory compromise due to diaphragmatic and intercostal muscle dysfunction [8]. Careful airway assessment is essential, accounting for cervical instability and limited neck mobility. Videolaryngoscopy, chosen by our team and recommended by several authors, is used to minimize cervical movement during intubation [8]. Awake fiberoptic intubation may be considered in selected cases. In the ICU, close respiratory monitoring, early tracheostomy when prolonged ventilation is anticipated, and preventive measures against complications such as pneumonia and atelectasis are crucial. Hemodynamic stability and careful patient positioning are also important for optimizing spinal cord perfusion and preventing secondary injuries. This multidisciplinary, individualized approach improves respiratory outcomes and overall prognosis in patients with spinal cord injury [8].

The comprehensive management, comprising early diagnosis, surgical drainage, targeted antimicrobial therapy, and hyperbaric oxygen, resulted in partial neurological recovery and successful extubation at two weeks, despite the high risk of unfavorable outcomes.

## Conclusions

SEA constitutes a rare yet potentially life-threatening condition associated with considerable morbidity and mortality. Early diagnosis, prompt utilization of MRI, and a multidisciplinary approach involving surgical source control and targeted antibiotic therapy are imperative to optimize patient outcomes. This case highlights the significance of considering SEA in patients presenting with risk factors such as intravenous drug use, especially when neurological deficits progress swiftly. Timely intervention is crucial to prevent permanent sequelae or death.
